# Thermoresponsive Azithromycin-Loaded Niosome Gel Based on Poloxamer 407 and Hyaluronic Interactions for Periodontitis Treatment

**DOI:** 10.3390/pharmaceutics14102032

**Published:** 2022-09-24

**Authors:** Kunchorn Kerdmanee, Thawatchai Phaechamud, Sucharat Limsitthichaikoon

**Affiliations:** 1 Department of Pharmaceutical Technology, College of Pharmacy, Rangsit University, Pathum Thani 12000, Thailand; 2 Department of Periodontics, College of Dental Medicine, Rangsit University, Pathum Thani 12000, Thailand; 3 Department of Pharmaceutical Technology, Faculty of Pharmacy, Silpakorn University, Nakhon Pathom 73000, Thailand

**Keywords:** periodontal pocket drug delivery, poloxamer 407, hyaluronic acid, periodontitis, thermoresponsive niosome gel

## Abstract

Azithromycin (AZM) is a potential antimicrobial drug for periodontitis treatment. However, a potential sustained-release system is needed for intra-periodontal pocket delivery. This study focused on the development and evaluation of a thermoresponsive azithromycin-loaded niosome gel (AZG) to search for a desirable formulation for periodontitis treatment. AZG was further developed from an AZM-loaded niosomal formulation by exploiting the advantages of poloxamer 407 (P407) and hyaluronic acid (HA) interactions. The results showed that the addition of HA decreased the gelation temperature and gelation time of AZG. HA was found to increase the viscosity as well as mucoadhesive and tooth-root surface adhesive properties. The AZG solution state was injectable and exhibited pseudoplastic shear-thinning behavior. P407–HA interactions in AZG could contribute to gel strength. AZG showed 72 h of continuous drug release following the Korsmeyer–Peppas model and potentially enhanced drug permeation. The formulations apparently presented more efficient antibacterial activity against major periodontal pathogens than the standard AZM solution. AZM intra-periodontal pocket formulation and the remarkable properties of niosomes exhibited potential characteristics, including ease of administration, bioadhesion to the anatomical structure of the periodontal pocket, and sustained drug release with competent antimicrobial activity, which could be beneficial for periodontitis treatment.

## 1. Introduction

Periodontitis is a destructive disease affecting the tooth-supporting structure or the periodontium. The reported prevalence of periodontal disease is 20–50% of the global population [1]. As described in the contemporary pathogenesis of periodontitis, the disease is initiated by the invasion of periodontal pathogens. When triggered by microbes, defensive host immunity retaliates via the destructive weapon known as the inflammatory process, which aims to remove microbes. Dysbiosis (the conflict between host and microbes) emerges and is aggravated by host environmental and genetic factors. Collateral damage is inevitable on every battlefield, and, in this context, periodontium results in the destruction of the tooth-supporting bone and periodontal pocket formation [2]. Severe periodontitis can cause multiple tooth losses, which deteriorates the patient’s quality of life. The gold standard of periodontitis treatment in the initial phase is mechanical debridement in conjunction with oral hygiene instruction [3]. However, this treatment modality yields limited outcomes, highlighting the need for potential adjunctive therapy [4]. The formation of the periodontal pocket is a clinical sign of periodontitis, which is the deepening of the gap between the tooth and gum. The depths of the periodontal pocket are difficult to adequately clean with self-care measures. Hence, the build-up of dental plaque biofilm containing pathogenic bacteria leads to the severe progression of periodontal disease. Although periodontal pockets are undesirable in maintaining health, this unique pathologic feature may be useful as a route for local drug delivery [5]. The active drugs could be directly delivered to the target site of the disease without passing through systemic metabolism. The local antibiotic application should avoid unwanted systemic side effects, such as nausea, vomiting, diarrhea, and bacterial resistance [6]. In the past decade, intra-periodontal pocket administration was utilized for various topical dosage forms.

Azithromycin (AZM) is a macrolide antibiotic with fascinating therapeutic properties. Apart from its susceptibility to major periodontal pathogens, its anti-inflammatory effects are well documented [7]. AZM’s dual effects could be beneficial for the pathogenesis of periodontal disease in respect to bacterial elimination and the modulation of the host inflammatory response. However, the poor solubility of AZM, which is in BCS class II, could affect its bioavailability in biological tissues [8]. Drug dissolution must be improved to enable the local delivery of AZM for intra-periodontal pocket administration. In our previous study, AZM was successfully prepared in a niosomal formulation to enhance its solubility, stability, and releasing properties [9]. With the advantages of niosomal vesicles, AZM could also be delivered through the lipid bilayer of periodontal tissues to eliminate the residing pathogens. For example, in periodontitis conditions, pathogenic microbes, such as *Aggregatibacter actinomycetemcomitans* (*Aa.*) and *Porphyromonas gingivalis* (*Pg*.), were not only colonized in the periodontal pocket but also infiltrated the subjacent connective tissue level of the gingiva [10,11]. Because a potential carrier for delivering AZM-loaded niosomes into the periodontal pocket is needed, a thermoresponsive azithromycin-loaded niosome gel (AZG) was formulated based on the interactions of poloxamer 407 (P407) and hyaluronic acid (HA). Injectable gel formulations are widely used in dentistry as various kinds of dental material. Dentists are familiar with this dosage form. Thus, the gel formulation could be effortlessly administered to patients. However, for the treatment of chronic diseases involving bacterial infections, such as periodontitis, formulation development should focus on the long retention time and sustained release of drugs at the active site. The formulation, which can transform into a semi-solid state, could be advantageous for this purpose because it tolerates the dynamic changes in the oral environment. Accordingly, P407, which exhibited phase transformation properties, was chosen. P407 is a thermoresponsive co-polymer with the ability to transform into a gel state upon increasing temperature. P407 is categorized as an inactive ingredient by the FDA and, because of its biocompatibility and biodegradability, is applied in various kinds of pharmaceutical formulations, including periodontal formulations [12,13]. Poloxamer-based hydrogels have well-documented low cytotoxicity and biodegradability [14]. Another crucial property that could contribute to the retention time is the adhesion of the formulation to biological tissues [15]. However, the drawbacks of P407 are low mucoadhesiveness, a weak hydrogel structure, and its rapid dissolution in water [16]. The imperfections of P407 can be corrected by the addition of other polymers.

HA is a naturally occurring biopolymer produced by hyaluronan synthase from the plasma membrane and is commonly found in the extracellular matrix of human epithelial and connective tissues. Therefore, as a member of the glycosaminoglycan family, HA has excellent biocompatibility and non-immunogenicity. Additionally, HA is readily biodegradable by hyaluronidases and oxidative species available in the human body [17]. HA possesses favorable mucoadhesive [18] and other beneficial properties for periodontal treatment, such as anti-inflammatory and accelerated wound-healing effects [19]. Consequently, HA was included in this formulation’s development. Recent studies showed that the coupling of P407–HA improved the textural integrity, rheological, and sustained drug release properties of the hydrogel matrix [15,20]. The selection of P407 and HA for AZM-loaded niosomes would favor the invention of a potential formulation with sustained antimicrobial activity for periodontitis treatment.

Therefore, the objective of this study was to develop a thermoresponsive azithromycin-loaded niosome gel for intra-periodontal pocket administration to improve the bioavailability of AZM in periodontal tissues. The physicochemical and mechanical properties of the formulations regarding the influence of P407–HA interactions were investigated to acquire a better understanding of the thermoresponsive gel based on P407 and HA. Drug release, mucosal permeation, and antibacterial studies were conducted to develop a desirable formulation for periodontitis treatment.

## 2. Materials and Methods

### 2.1. Materials

Azithromycin (AZM) was provided as a gift from Siam Chemi-Pharm (1997) Co., Ltd., Bangkok, Thailand. Poloxamer 407 (P407), cholesterol (CHL), Span^®^ 60 (S60), Nile red (9-diethylamino-5H-benzo[alpha]phenoxazine-5-one), type II mucin from the porcine stomach (Sigma-Aldrich, St. Louis, MO, USA), and sodium hyaluronate (HA) 2.05 × 10^6^ Da MW (SpecKare™, Nanjing, China) were purchased from local suppliers and used as received.

### 2.2. Preparation of Thermoresponsive Niosome Gel

AZG formulations were further developed from our previous study of AZM-loaded niosomes [9]. The niosomal suspension of AZM was fabricated by entrapping it into the niosomes of S60 and CHL utilizing the modified reverse-phase evaporation method. The amounts of S60 and CHL at the molar ratio of 3:3 (0.42 and 0.38 g, respectively, in the preparation of 30 mL niosomal suspension) and 1% of AZM were dissolved with absolute ethanol and submerged in an ultrasonic bath (POWERSONIC CP230T, Crest Ultrasonics, Ewing Township, NJ, USA) for 30 min. Then, deionized water as a secondary solvent was added and the mixture was continuously ultrasonicated for 30 min. After a homogeneous mixture was obtained, ethanol was eradicated by a rotary evaporator (N-1001, Eyela, Tokyo, Japan). The niosomal suspension was kept at 4 °C for 24 h to allow vesicle maturation. The niosomes were dyed with Nile red and we observed the vesicle staining under a confocal laser scanning microscope (DMi8, Leica, Wetzlar, Germany). Afterward, the thermoresponsive niosome gel formulations were prepared by adding HA and homogeneously mixing them with a propeller stirrer at 200 rpm (RW 20 digital, IKA, Staufen, Germany). Then, P407 was incorporated into the formulations by the cold method. Accurately weighed amounts of P407 were gradually added to the pre-mix of AZM-loaded niosomes and HA, which had been equilibrated at 4 °C. The amounts of HA and P407 varied, as presented in Table 1.

The mixtures of AZG formulations were stored overnight at 4 °C. The formulations were intermittently stirred until uniform mixtures were acquired. The pH of each formulation was examined using a digital pH meter (Eutech pH 700, Eutech Instruments Pte Ltd., Singapore). The prepared formulations were maintained at 4 °C in sealed containers.

### 2.3. Drug Content

To determine the total AZM content in the prepared formulations, one mL of each formulation was pipetted into a 10 mL volumetric flask. The volume was composed of methanol as the extraction solvent. The solution was left for 24 h. Then, the gel formulation was completely dissolved, which produced a clear solution. The extracted samples of each formulation were quantitatively investigated for drug content by a modified HPLC method (n = 3) [21]. The analysis was carried out on an HPLC system (LC-10, Shimadzu, Kyoto, Japan) equipped with a C18 column (Zorbax Eclipse XDB-C18, Agilent Technologies, Santa Clara, CA, USA). The mobile phase was composed of 80% of MeOH and 20% of 0.3 M KH_2_PO_4_ pH 7.56, under the conditions of a 1 mL/min flow rate, 50 °C, and 210 nm of the diode array detector. The injection volume was 20 µL.

### 2.4. Gelation Temperature

The phase transition temperature of the prepared thermoresponsive formulations was determined using a Brookfield viscometer (DV-II+ viscometer, Brookfield Engineering Laboratories, Middleborough, MA, USA). A sample container connected with a temperature-controlled jacket was filled with the formulations. The viscometer probe was set at 10 rpm, a fixed rotational speed, to measure the alteration in viscosity. The temperature of the system was controlled to increase from 3 °C to 40 °C, while the viscosity of the formulation was monitored. A significant elevation in viscosity at a specific temperature was observed and recorded as the gelation temperature of each formulation (n = 3).

### 2.5. Gelation Time

The time duration that the formulation needed for phase transformation from solution to gel state was examined by the test tube inversion method. Thin-walled scintillation glass vials were filled with the formulations and submerged in a temperature-controlled water bath, which was set to 37 °C. The phase transformation was visually observed. The total time needed for the meniscus of the formulation to stop moving upon tilting was recorded as the gelation time of each formulation (n = 3).

### 2.6. Gel Viscosity

The viscosity of the prepared formulations was evaluated using a Brookfield viscometer (DV-II+ viscometer, Brookfield Engineering Laboratories, USA). The sample container was connected to a temperature-controlled jacket. The viscometer was set at 10 rpm, a fixed rotational speed. The measurements of the solution and gel states of the formulation were conducted at 4 °C and 37 °C, respectively (n = 3).

### 2.7. Injectability of the Formulations

The prepared formulations were loaded into 1 mL syringes with a 22-gauge stainless-steel needle (0.7 mm diameter) intended for use in clinical situations. The syringe was fixed with a vertical holder aligned at the base platform of the texture analyzer (TA.XT PlusC, Stable Micro Systems, Surrey, UK). A cylindrical probe (Model P/0.5, 12.7 mm diameter) was directed downward to push the plunger rod of the syringe at a speed of 10 mm/s. The maximum force applied to inject the formulation from the syringe barrel through the needle tip was measured. The measurements were conducted immediately after each formulation was brought from the refrigerator, while the formulation was in the solution state (4 ± 5 °C).

### 2.8. Rheological Study

The rheological behavior of the solution state AZG was determined using the Kinexus pro rheometer (Malvern Instruments Ltd., Worcestershire, UK) with a PL20 stainless-steel parallel plate (20 mm diameter). The temperature during measurement was controlled at 4 °C. Shear stress was measured as a function of shear rate (n = 3), which varied from 0.1 to 100 s^−1^, and the obtained data were analyzed by rSpace Rheometry software for Kinexus version 1.75.2326. Flow profiles were fitted with various rheological equations, such as the Newtonian (Equation (1)), power law (Equation (2)), Bingham (Equation (3)), Hershel–Bulkley (Equation (4)), and Casson models (Equation (5)).

*τ* = *ηγ* (1)

*τ = K·γ ^n^* (2)

*τ = τ*_0_*+ η_p_·γ* (3)

*τ = τ*_0_*+ K·γ ^n^* (4)

*τ*^0.5^*= τ*_0_^0.5^ + *K*·*γ*
^0.5^ (5)

where *τ* is shear stress, *η* is viscosity, *γ* is the shear rate, *η_p_* is plastic viscosity, *K* is the consistency index, *τ*_0_ is the yield value, and *n* is the flow index.

### 2.9. Mucoadhesive Property

The mucoadhesion of each formulation was evaluated using the mucin disc model, with slight modifications [22,23]. The mucoadhesive force was measured with a texture analyzer (TA.XT PlusC, Stable Micro Systems, Surrey, UK). The mucin discs were prepared with 250 mg of crude mucin powder by utilizing a 13-mm-diameter die with the vacuum ring compression set at 10 tons for 30 s. One mucin disc was attached at the center of a 60 mm Petri dish, which was held to the base platform of the texture analyzer. Another mucin disc was attached to the cylindrical probe tip of the texture analyzer while the formulation was applied between the two mucin discs at 0.1 mL. The probe was moved downward until a 1 mm space between the discs was reached. The formulation was induced to form a gel state. Subsequently, the probe was directed to compress and held for 30 s, before moving upward at a rate of 10 mm/s. The maximum force used to separate the mucin discs from each other was measured as the mucoadhesion force of each formulation (n = 3).

### 2.10. Tooth-Root Surface Adhesion

Tooth-root surface specimens were prepared from extracted human teeth, with slight modifications from the previous study [23]. The flat-surface roots were equally sectioned into 6.6 × 6.6 mm pieces with a thickness of 1.5 mm, with a micro motor (Strong 90, Saeshin, Daegu, Korea) equipped with a diamond cutting disc. Three of the root specimens were attached to a 25 × 25 mm acrylic plate with cyanoacrylate glue (UHU Super Glue, UHU GmbH & Co. KG, Bühl, Germany). The acrylic plate was fixed at the center of a 60 mm Petri dish, which was held at the base platform of the texture analyzer. Three more root specimens were prepared in the same way and attached to an identical acrylic plate. The second acrylic plate was attached to the cylindrical probe of the texture analyzer in the same alignment. The formulation was applied between the tooth-root surface specimens. The tooth-root surface adhesive force of each formulation was evaluated by the texture analyzer in the same manner as in the mucoadhesive study (n = 3).

### 2.11. Texture Profile Analysis

The gel state of the prepared formulations was investigated for texture profile properties by the texture analyzer (TA.XT PlusC, Stable Micro Systems, Surrey, UK). Each formulation was loaded in a 55 mm culture dish and induced to form a gel state. The double compression method was utilized [23]. The probe of the texture analyzer was moved downward at a speed of 2 mm/s until it contacted the surface of the gel formulation. Then, the probe was directed to penetrate the gel matrix for half of its height, and the probe was withdrawn upward to the first surface-contact position. The probe was held at this position for 15 s before running the second compression in the same manner as the first. The hardness, springiness, and resilience values of each formulation were calculated from the force–time graphs provided by the texture analyzer software (n = 3).

### 2.12. In Vitro Drug Release Study

The drug release characteristics of the prepared formulations were investigated with the Franz diffusion cell apparatus. The dialysis membrane with a 12 kDa MW cutoff (Sigma-Aldrich, St. Louis, MO, USA) was utilized. The surface area of the diffusive interface was 176.625 mm^2^. One mL of each formulation was accurately pipetted and applied to the donor compartment. The receptor compartment (11 mL volume) was filled with the dissolution medium, which was a phosphate buffer of pH 6.8, and magnetically stirred at 300 rpm. The system temperature was controlled at 37 °C. Samples of the medium containing the released drug were withdrawn for 1 mL at predetermined time points and refilled with the equivalent volume of fresh medium. The sink condition was maintained throughout the experiment. The quantities of released AZM in each sample were analyzed using the HPLC method, as previously described. The drug release profiles were plotted and mathematically fitted with kinetic models—for example, the zero-order (Equation (6)), first-order (Equation (7)), Higuchi (Equation (8)), and Korsmeyer–Peppas models (Equation (9)).

*Q_t_ = K*_0_·*t* (6)

ln *Q_t_* = ln *Q*_0_ − *K*_1_·*t* (7)

*Q_t_* = *K_H_·t*^1/2^ (8)

*D_t_/D_∞_ = K_KP_·t^n^* (9)

where *Q_t_* is the amount of drug released at time *t*; *Q*_0_ is the initial amount of the drug in the formulation; and *K*_0_, *K*_1_, and *K_H_* are the release rate constants of the zero-order, first-order, and Higuchi models, respectively. In Equation (9), *D_t_/D_∞_* is the proportion of drug released at time *t*, *K_KP_* is the kinetic constant, and *n* is the release exponent.

### 2.13. Ex Vivo Permeation Study

Mucosal permeation behavior was observed using the Franz diffusion cell apparatus method. Porcine esophagus mucosa was used as a permeation membrane [24]. Fresh porcine esophagi of comparable size and appearance were purchased from the local slaughterhouse. The esophagus was resected to remove the muscle and excised to obtain mucosal specimens with sizes of 30 × 30 mm and with 2 mm thickness. The epithelium side of the mucosal membrane was positioned facing the donor compartment, whereas the connective tissue side was positioned facing the receptor compartment. The diffusive surface area was 176.625 mm^2^. The receptor compartment was fully filled with 11 mL of phosphate buffer of pH 6.8 as a dissolution medium. The donor compartment was loaded with 1 mL of AZG formulation. The system was continuously stirred at 300 rpm at 37 °C. One mL of medium sample was collected at predetermined time points and refilled with an equal amount of fresh medium. The sink condition was maintained. Permeated drugs in the sample were quantified with HPLC, as previously mentioned (n = 3).

### 2.14. Antibacterial Studies

AZG formulations were evaluated for an antibacterial assay against *Aggregatibacter actinomycetemcomitans* (*Aa.*, ATCC 43718) and *Porphyromonas gingivalis* (*Pg.*, ATCC 33277) using the agar well diffusion method. The lyophilized bacterial strains were grown in tryptic soy broth (TSB, Himedia Laboratories, Mumbai, India) for 36 h at 37 °C in an anaerobic jar with GasPak (Becton, Dickinson, and Company, Sparks, MD, USA). The organism turbidity of the broth suspensions was checked using the 0.5 McFarland standard. The standardized inocula of *Aa*. and *Pg*. were prepared at the concentration of 1.5 × 10^8^ cells/mL and swabbed on the surface of sheep blood agar (Medex Solutions Ltd., Saraburi, Thailand). A sterile cork borer was used to create an 8-mm-diameter well on the inoculated agar before introducing 0.1 mL of formulation to the well. The equivalent concentration of AZM in a phosphate buffer pH 6.8 solution-soaked disc was used for comparison. The tested samples were incubated in an anaerobic incubator for 24 h. Then, the inhibition zone of each formulation was measured (n = 3).

### 2.15. Statistical Analysis

All measurements were performed in a triplicate manner. All categorical variable data were evaluated as percentages (n = 3). Continuous variable data are described as mean and standard deviation (SD). A *p*-value of <0.05 was taken as statistical significance and analyzed using SPSS 13 software (SPSS Inc., Chicago, IL, USA).

## 3. Results and Discussion

### 3.1. Formulation Preparation

**Figure 1:**
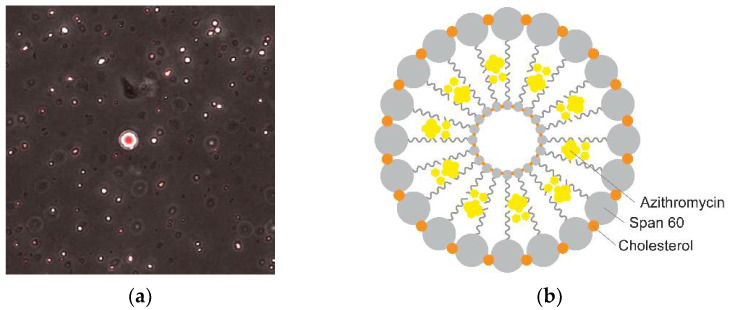
(**a**) Visualization of prepared AZM-loaded niosomes stained with Nile red dye that indicated lipid droplet as a red-stained vesicle under confocal laser scanning microscope; (**b**) schematic illustration of AZM-loaded niosomes prepared from cholesterol and Span^®^ 60 (adapted from Moghassemi and Hadjizadeh 2014 [25]).

AZM is a potential drug for periodontitis treatment [7]. However, because of its poor water solubility, it is classified as a BCS class II drug. This may affect the bioavailability of AZM in periodontal tissue for a localized dosage form. Therefore, an appropriate delivery system is needed. In this investigation, a niosome template was applied to formulate an efficient carrier for AZM. Practically, the niosomal vesicle system increases the solubility of hydrophobic drugs, enhances drug permeation, and improves the stability of the formulation [25,26]. AZM niosomes were prepared based on the modified reverse-phase evaporation method. AZM was successfully loaded into niosomes with nano-sized particles, charge stability, and biocompatibility [9]. Figure 1a presents confocal images of Nile-red-stained unilamellar niosomal vesicles obtained by confocal laser scanning microscopy. The hypothesized niosome vesicle structure is displayed in Figure 1b.

The vesicle consists of a double layer of S60, which is stabilized by CHL [25]. AZM was entrapped within the hydrophobic tails of S60. In this work, AZM-loaded niosomes were further developed into the form of a thermoresponsive gel for intra-periodontal pocket administration in clinical applications. To study the physical interaction of P407 and HA with the niosomal formulation, AZG was successfully prepared into nine different formulations by varying the concentrations of P407 (17–19% *w*/*v*) and HA (0.2–2% *w*/*v*) (Table 1). The prepared formulations appeared as a homogenous white, opaque solution owing to the appearance of the niosomal suspension prepared from CHL and S60. All formulations remained in uniformed mixtures that were not reliant on the concentrations of P407 and HA.

The measured pH of all prepared formulations was in the range of 6.89 ± 0.01 to 6.92 ± 0.04 (Table 2). The pH of the AZG formulation was slightly reduced from that of the prepared AZM-loaded niosomes, which were 7.04 ± 0.06. In periodontitis conditions, the pH of the periodontal pocket could decrease from the mean value of 6.92 ± 0.03 [27]. Therefore, the prepared formulations should be compatible with the periodontal pocket environment, without causing irritation to the biological tissues of the patient. Drug content data revealed the percentage of loaded AZM as ranging from 93.09 ± 0.94 to 94.49 ± 0.81 (Table 2). The drug content of AZG regarded the entrapment efficiency of AZM-loaded niosomes.

### 3.2. Thermoresponsive Properties

AZG was designed to convert from solution to gel state after being injected into the periodontal pocket, which accounted for the addition of P407 to the composition. The phase transformation of the formulations was evaluated in terms of gelation temperature and gelation time (Table 2). The average gelation temperature varied from 27.83 ± 0.5 to 40.33 ± 0.06 °C. In the formulation groups with an equal amount of HA, the influences of P407 were observed. The incremental increase in P407 concentration significantly reduced the gelation temperature (*p* < 0.01). P407 is a triblock co-polymer composed of double hydrophilic polyethylene oxide (PEO) sandwiching with single hydrophobic polypropylene oxide (PPO) in between. Upon temperature increase, PPO groups interact with each other with van der Waals forces and form hydrophobic cores, while PEO groups build up the hydrophilic shells of micelles with hydrogen bonds to the water molecule [12]. Upon further temperature increases, micelles aggregate at a certain temperature and arrange into 3D cubic forms to achieve gel-state transformation. The increase in P407 concentration resulted in the abundance of co-polymers to facilitate micelle formation [28].

Gelation time, which represents the setting time of the formulation, was in the range of 68.00 ± 2.00 to 227.00 ± 3.61 s. Similar to the gelation temperature, the increase in P407 concentration from 17 to 19% significantly reduced the gelation time (*p* < 0.01). However, AZG1 was unable to form a gel state because the gelation temperature was higher than 37 °C. In the formulation groups with an equal amount of P407, the increase in HA concentration from 0.2 to 1.1% significantly reduced the gelation temperature (*p* < 0.01). The increase in HA from 1.1 to 2% showed no significant changes in the gelation temperature, except in the group with 19% of P407, which exhibited a significant reduction in the gelation temperature (*p* < 0.01). Moreover, the increase in HA concentration was found to significantly decrease the gelation time (*p* < 0.01). However, in the group with 2% of HA, the increase in P407 concentration from 17 to 18% only showed a trend of reduction.

The addition of HA in the formulation exhibited the tendency to reduce the AZG gelation temperature and gelation time. This phenomenon could be explained by the incorporation of high-molecular-weight HA caused by the high-density packing of HA and P407 molecules, which could facilitate the micellization process of P407 [29]. Moreover, the additives in the P407 formulation, which could form non-covalent bonds with P407, reduced the gelation temperature by decreasing the interaction of P407 with water [12,28]. In this situation, the availability of the hydroxyl and carboxyl groups of HA could form hydrogen bonds with P407 [20]. This resulted in dehydration, which could facilitate the micellization process because there were fewer water molecules to interfere with the joining of PPO in the hydrophobic cores of the micelles [30]. Another possible explanation could be that the strong hydrophilicity of HA, a powerful humectant, attracted the water fraction from the molecular chain of the poloxamer, contributing to the reduction in gelation time [31]. There are many phase transformation mechanisms that could be utilized in formulation development, such as pH, temperature, and solvent exchange. Among others, temperature is a common physiological state of the human body, and it may be regarded as the simplest triggering mechanism. The prepared formulation would readily perform phase transformation at the active site. In clinical practice, the dosage-form setting time should not take too long; otherwise, the formulation might prematurely dislodge from the periodontal pocket.

### 3.3. Viscosity and Injectability

The viscosity of all formulations is displayed in Table 2. At 4 °C, the formulations were in the solution state. The measured viscosity was in the range of 2.24 ± 0.25 to 143.53 ± 15.55 cps. The viscosity was found to increase significantly upon the increase in HA concentration from 17 to 18% (*p* < 0.05) and 18 to 19% (*p* < 0.01). On the other hand, the increase in P407 showed no significant changes in the viscosity as no micellization occurred.

At 37 °C, all formulations transformed into the gel state. The viscosity was elevated to the range of 162.77 ± 5.75 to 283.77 ± 3.75 cps. The increases in HA concentration were found to significantly enhance the viscosity of the gel-state AZG (*p* < 0.05). Except in the group of 17% P407, the changes in HA from 1.1 to 2% only presented an increasing trend. The influence of P407 indicated that the increase in P407 concentration from 17 to 18% showed an increasing trend but was not statistically significant, whereas the increase from 18 to 19% exhibited significantly elevated gel-state viscosity (*p* < 0.05). The higher viscosity in each formulation was influenced by the increased concentration of both P407 and HA in the formulation. Phase transformation of P407 via micellization directly affected the gel-state viscosity. The influence of HA on the increase in viscosity was clearly present in the formulation solution state as a result of the high molecular weight of HA included in AZG (2.05 × 10^6^ Da).

Then, the solution state of all formulations was evaluated for injectability (Table 3). The force used to inject the formulation showed a similar trend to the viscosity of each formulation. However, the maximum force used to expel the formulations through the needle tip of the syringe was less than 2 N in all formulations. Therefore, all formulations were considered injectable. This should facilitate intra-periodontal pocket administration. Thus, clinicians could conveniently inject the formulation into the narrow space of the periodontal pocket with minimal pressure.

### 3.4. Rheological Behavior

**Figure 2:**
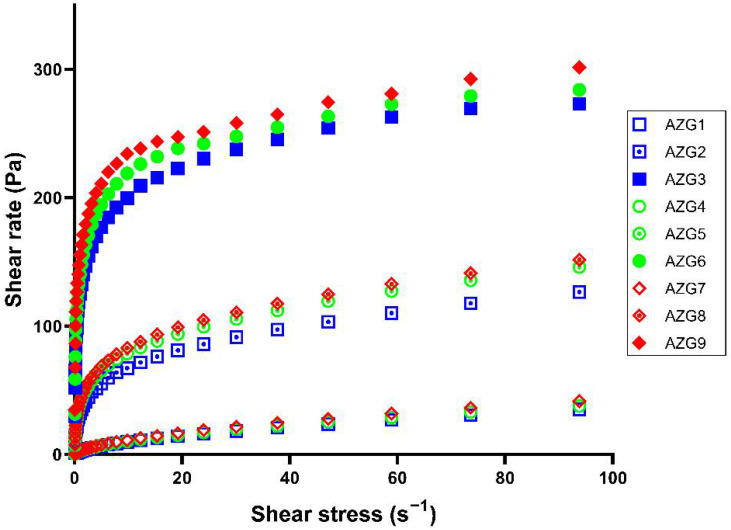
Shear stress and shear rate plots indicating rheological behavior of the solution state of AZG formulations (mean value).

The AZG solution state was investigated for flow behavior. The shear stress and shear rate curves of the prepared formulations are displayed in Figure 2. All AZG formulations exhibited a non-Newtonian fluid flow according to the nonlinear relationship between shear stress and shear rate. The slope of the curves indicated that the viscosity of the AZG formulations decreased upon the shear force applied, which demonstrated pseudoplastic behavior [32]. The viscosity and shear stress increases are attributed to the increased concentrations of P407 and HA in the formulation.

When the plots were analyzed with rheological equations, it was discovered that rheological data were best fitted with the Herschel–Bulkley model, which is typical for non-Newtonian fluids. The flow index (n value) from the Herschel–Bulkley equation of all formulations was in the range of 0.189 ± 0.003 to 0.585 ± 0.023, which indicated shear-thinning flow behavior (n value < 1). Regarding the injectable dosage form, pseudoplastic behavior was considered desirable. Force is needed for pseudoplastic fluids to flow. When the shear force was applied to the formulation, the entangled molecular structure changed and was oriented toward the direction of the force [33]. Then, the formulation could flow through the syringe barrel and the needle tip to the target site. After injection, the formulation could spread into the complicated anatomical structure of the periodontal pocket by the injection force, which reduced the viscosity of the pseudoplastic formulation. After thoroughly spreading into the target site, the formulation should reinstitute its viscosity and remain in the periodontal pocket without dripping due to the pseudoplastic shear-thinning effect [34].

### 3.5. Bioadhesive Properties

Aside from the phase transformation feature, the adhesion of the formulation to the biological tissues extends the retention time of the formulation. Based on histopathology, one side of the periodontal pocket is bordered by the pocket epithelium and the other side is the tooth-root surface, and the junctional epithelium is located at the base of the pocket [35]. In this study, the bioadhesive properties of AZG were examined for both sides of the periodontal pocket territory, which involved mucoadhesion to the pocket epithelium and the adhesion of AZG to the tooth-root surface.

#### 3.5.1. Mucoadhesion

**Figure 3:**
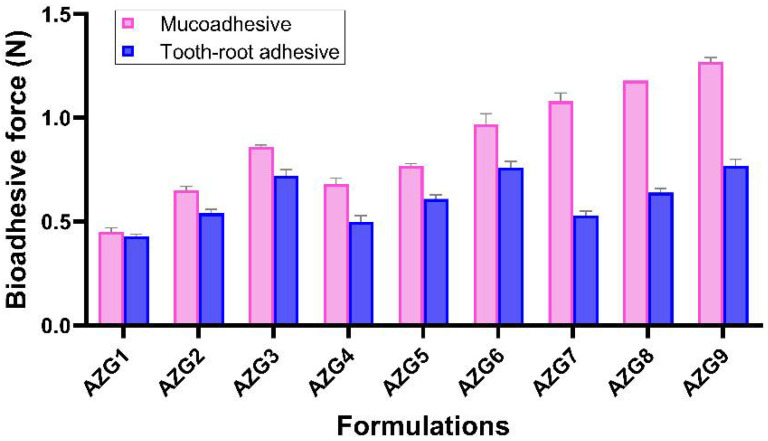
Bioadhesive force of AZG formulations representing adhesion to mucosa and tooth-root surface (mean ± SD, n = 3).

The evaluation of the mucoadhesive properties of all AZG formulations is displayed in Figure 3. The mucoadhesive force was in the range of 0.45 ± 0.02 to 1.27 ± 0.02 N. A higher concentration of HA in the formulation resulted in a significantly greater mucoadhesive force (*p* < 0.01). In the formulation with the same amount of HA, the higher amount of P407 also significantly increased the mucoadhesive force of each formulation (*p* < 0.01). This illustrates the synergistic effect between P407 and HA on mucoadhesive enhancement. Despite being a versatile excipient, the bioadhesion of P407 alone was weak, which could affect the potential of topical formulations [16]. In poloxamer-based formulations, additives are needed in order to improve the mucoadhesive properties. In this regard, HA can be employed as a mucoadhesive biopolymer, which can help to improve the mucoadhesive properties in various dosage forms [36,37,38]. HA mucosal bonding can be explained with mucoadhesive theories. First, the HA molecular structure results in hydrogen bonding with biological surfaces [39]. Second, the HA coil structures entangle the mucous membranes [40]. High-molecular-weight HA possesses multiple coil structures, which accommodate the entanglement process. Therefore, high-molecular-weight HA exhibited higher mucoadhesive force than low-molecular-weight HA [18].

In this study, the synergistic effects of P407 and HA in mucoadhesion were observed. The surfactant role and the hydrophilicity of P407 during the solution state could facilitate the penetration and entanglement of HA into the mucoadhesive interface and set up stronger adhesion [41]. The oral environment is a challenging condition for dosage-form development due to the various dynamic changes, such as saliva flow and the movement of the tongue and mastication muscles. In addition, the formulation for intra-periodontal pocket administration must further withstand the gingival crevicular fluid (GCF) flow, which is secreted from the junctional epithelium at the bottom of the periodontal pocket. The reported flow of GCF is 0.33–0.5 µL/min, which tends to flush out the periodontal pocket [42]. Therefore, a formulation with high bioadhesive properties could overcome these unfavorable oral environment conditions.

#### 3.5.2. Tooth-Root Surface Adhesion

The adhesive force of AZG on the tooth-root surface specimens was in the range of 0.43 ± 0.01 to 0.77 ± 0.03 N (Figure 3). The increase in HA concentration resulted in significantly higher adhesion (*p* < 0.01). An increase in P407 was only found to significantly enhance tooth-root adhesion in the group with 0.2 and 1% of HA; the increment in P407 from 17 to 18% significantly increased the adhesion (*p* < 0.05), while the other groups showed no significant statistical differences upon varying the P407 concentrations. In contrast to the mucoadhesive study, the results revealed that the adhesion to the tooth-root surface was mainly dependent on the concentration of HA. The mechanism of adhesion to the tooth-root surface can be explained by mechanical theory. The mucoadhesive substance filled and adhered to the rough or irregular surfaces with an increased contact area of the adhesive interface [43].

The root surface of the human tooth is covered by cementum, which is porous in structure. The formulation adhesiveness was possibly improved by penetrating and entangling the coil structures of HA into the microporosity of the root surface. In clinical situations, mucin from saliva infiltrates the root microporosity, which would further aid in the formulation’s adhesion. Formulation adhesion to the tooth structure is currently being researched. According to the histopathologic features of the periodontal pocket, the pocket lining epithelium was ulcerated due to the inflammatory stage of periodontitis. The repairing epithelium was turned over at a high rate, and the shedding of the fragile epithelium layer could remove the adhered formulation from the periodontal pocket [23]. Therefore, the adhesion to the tooth-root surface prevents formulation dislodgement. Increased HA concentrations apparently increase the AZG formulation’s tooth-root surface adhesion.

### 3.6. Texture Profile Analysis

Texture profile analysis is widely used in food sciences for characterizing textural properties, such as hardness, springiness, and resilience. These properties are also useful in the pharmaceutical technology field. Thus, many recently published studies adopted this technique to analyze invented dosage forms [22,23,30]. Gel-state AZG was investigated for its textural properties to understand the gel behavior during residence in the periodontal pocket (Table 3). Hardness is the maximum force of the first compression to penetrate the gel formulation [44]. It was discovered that the increase in HA significantly increased the gel-state hardness (*p* < 0.01). When determining the influence of P407, it was found that the increase in the concentration of P407 significantly increased the gel hardness (*p* < 0.05). However, in the 0.2 and 2% HA group, the increase in P407 from 17 to 18% only showed an increasing trend of hardness but was not statistically significant. The results indicated that the hardness of the gel state was directly dependent on the formulation’s concentration of P407 and HA. Hardness represents the strength of the gel matrix, which was in the range of 249.80 ± 6.90 to 617.29 ± 14.31 mN. The gel strength of all prepared formulations was comparable to other periodontal formulation studies [23,30]. Although P407 could perform phase transformation, the obtained gel matrix is limited in its pharmaceutical application due to structural weaknesses and its rapid dissolution in water. The P407 matrix gel strength could be improved by the addition of a second polymer, or by the modification of its chemical structure [16].

In this study, the AZG matrix was reinforced by the addition of HA. The coupling of P407 and HA resulted in P407–HA interactions, which occurred by secondary bonds, such as hydrogen bonds, and the formation of large micelles embedded into the coil structures of HA, which improved the rheological properties of the hydrogel matrix [20]. The stronger matrix could be effectively maintained in the periodontal pocket. However, the gel should not be too hard, which would allow deformity inside the periodontal pocket. Otherwise, the gel state may hinder the periodontium repair process. Additionally, springiness is how well a product physically “springs back after it has been deformed”. Resilience is how well a product “fights to regain its original height” [44]. It was found that springiness was in the range of 0.24 to 0.25, while resilience was low and in the range of 0.002 to 0.003. The data indicated that the gel state of all prepared formulations exhibited no bounce-back behavior. Increased P407 and HA concentrations showed no influence on the springiness and resilience. These results were in accordance with previous textural studies [23]. The AZG gel state should be able to deform within the periodontal pocket. Therefore, AZG should not interfere with periodontal tissue growth during the healing process.

All AZG formulations prepared in this study were able to perform phase transformation at physiological temperatures using different gelation times. These formulations could be used for intra-periodontal pocket administration. However, when considering its clinical application as a dental material, the long setting time would be impractical. In terms of viscosity and rheology, the formulations that had low viscosity and pseudoplastic properties could be difficult to manipulate during injection because of the free-flowing fluids. The formulations with moderate viscosity could facilitate administration by injection, enabling clinicians to control the volume of the formulation administered. In terms of the rheological aspect, the moderate-viscosity formulation needs a higher injection force, which should help to push the formulation to efficiently penetrate through the narrow gap of the soft tissue inside the periodontal pocket. Furthermore, the formulations that exhibited high mucoadhesive and tooth-root adhesion forces could potentially achieve a long retention time and sustained drug release within the periodontal pocket. Therefore, considering all the above-mentioned factors, AZG3, 6, 8, and 9 were high-potential formulations and were chosen for further examinations.

### 3.7. In Vitro Drug Release and Kinetic Profiles

**Figure 4:**
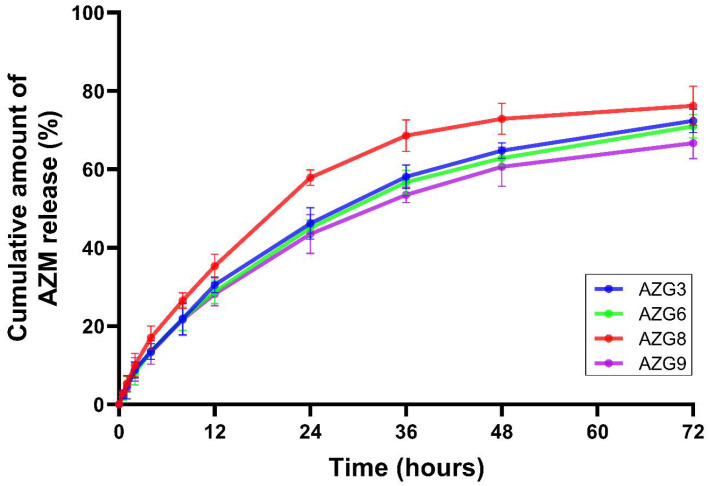
Percent cumulative drug release of AZG formulations. Data are mean ± SD, n = 3.

The cumulative release plots of AZM for 72 h from AZG3, 6, 8, and 9 are displayed in Figure 4. The release profiles of the four formulations were similar in behavior and there was no lag time presented in all formulations. It was found that AZG8 exhibited the highest drug release rate, followed by AZG3, 6, and 9. In the fourth hour, AZG8’s release considerably increased, becoming higher than others, and continued for 72 h. The cumulative release plots differentiate the release profile into three phases: (1) fast release at the first 12 h; (2) sustained release from 12 to 48 h; and (3) steady state of release at 48–72 h [45]. The development of AZG formulations for periodontitis treatment was expected to feature fast drug release after administration, and then maintained release, which would achieve antimicrobial activity inside the periodontal pocket for 72 h according to the AZM oral administration dosage [7]. Data from the cumulative plots revealed that all tested formulations exhibited similar release profiles with prompted and sustained AZM release for up to 72 h. However, AZG8 presented the highest release rate compared with other formulations.

The cumulative drug release data were analyzed with various kinetic models, such as the zero-order, first-order, Higuchi, and Korsmeyer–Peppas models. The results of linear regression with the equation parameters of each formulation are displayed in Table 4. The AZG formulation release rates were best fitted to the Korsmeyer–Peppas model, which indicated that the AZG formulation drug release was provided by polymeric matrix drug delivery [46]. When considering the release exponent (n) of the Korsmeyer–Peppas equation, the n-values were in the range of 0.45 < n < 0.89 [47]. Therefore, the drug is released in a non-Fickian diffusion manner. AZM formulation release was from both polymeric matrix diffusion and erosion [48]. The physical properties of AZG played an important role in the release behavior. AZG8, which yielded the highest release rate, consisted of 18% P407 and 1.1% HA. Decreased concentrations of P407 and HA resulted in lower hardness in the tested group. Consequently, the AZG8 gel matrix easily eroded, allowing faster drug release. A similar explanation could be applied to the releases of AZG3, 6, and 9. These formulations were equally composed of 2% HA, and the hardness of the gel matrices was solely dependent on the concentrations of P407. AZG3, with 17% P407, had the lowest gel hardness compared with AZG6 (18% of P407) and 9 (19% of P407). Therefore, AZG3 exhibited the highest drug release rate, followed by AZG6 and 9.

As mentioned in the texture profile analysis, the interactions between P407 and HA created a stronger hydrogel matrix [20]. The P407–HA hydrogel matrix could be used as a controlled-release device to sustain and slow the release of drugs because of its improved structural integrity and stability [15]. Regarding the AZM oral regimen, 500 mg is provided orally once a day for 3 days, which is considered a short course of administration [7]. Therefore, the efficient sustained-release system would be able to deliver AZM with a single application. This should encourage patient compliance and would potentially reduce dental visits for periodontal treatment, especially during the COVID-19 pandemic.

### 3.8. Ex Vivo Permeation Study

**Figure 5:**
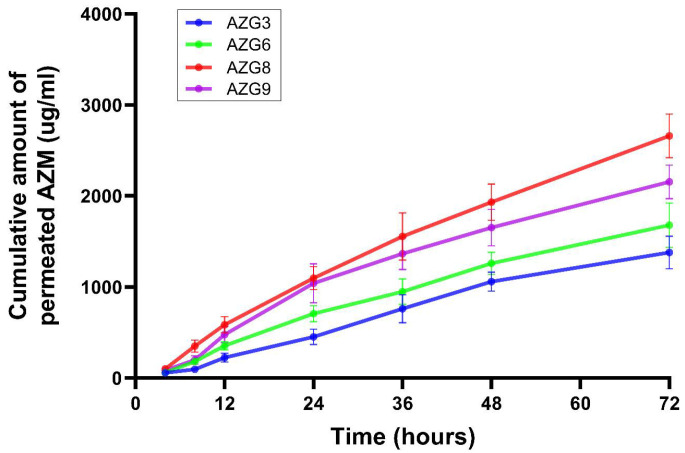
Cumulative permeated drug from ex vivo mucosal permeation model. Data are mean ± SD, n = 3.

The amount of AZM permeated through the mucosal specimen is displayed in Figure 5. The permeated AZM was highest in AZG8, owing to its lowest HA loading compared with other tested formulations, followed by 9, 6, and 3. Therefore, the obtained molecular structure was loosely packed and combined with low textural hardness, and was also prone to erosion. Moreover, AGZ8’s high mucoadhesion also contributed to the high permeation rate due to its formation of a strong mucoadhesive interface, which adhered closer to the mucous [49]. When making comparisons between AZG3, 6, and 9, which had equivalent amounts of HA (2%), AZG9 with 19% of P407 showed the highest permeation rate, followed by 6 (18% of P407) and 3 (17% of P407). The P407 concentration could be responsible for the high permeation rate. It was found that P407 increased transmucosal drug delivery [50]. Apart from this, AZG9 also possessed the highest mucoadhesive properties.

In this study, the porcine esophagus was utilized as a mucosal permeation model because of its structural properties that resemble the human oral mucosa. The preparations to obtain the standardized esophageal specimens were uncomplicated compared with the porcine buccal mucosa, which might be destroyed by mastication, are varying in texture, and have limited availability [24]. The log P value of AZM, which is 3.98, indicated that AZM is limitedly soluble in water and tends to disperse in lipids [51]. This suggests that the low number of permeated drugs was due to AZM being mostly distributed in the lipid bilayer of the mucosal specimen. This situation would be beneficial for periodontitis treatment. As a role of topical formulation, it was expected that the formulation would provide slow drug release within the periodontal pocket, deliver the drugs permeating through the periodontal pocket epithelium, and then maintain the drugs within the epithelium and connective tissue of the gingiva to eliminate the infiltrated periodontal pathogens.

### 3.9. Antibacterial Activity

**Figure 6:**
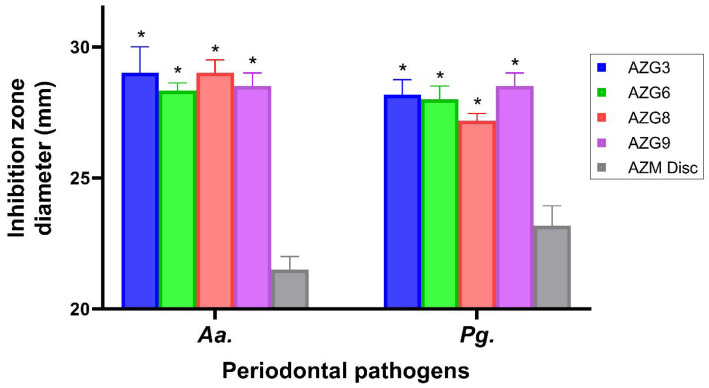
Antibacterial activity of AZG against major periodontal pathogens, namely *Aggregatibacter actinomycetemcomitans* (*Aa*.) and *Porphyromonas gingivalis* (*Pg*.). Data are mean ± SD, n = 3. Asterisks indicate statistically significant differences compared with AZM disc (*p* < 0.01).

AZG formulations were evaluated for antibacterial properties against major periodontal pathogens, namely *Aa.* and *Pg.* (Figure 6).

The results of the inhibition zone indicated that all AZG formulations exhibited significantly higher antibacterial activity (*p* < 0.05) than the AZM solution at the equivalent concentration. This might be due to the hydrogel matrix of AZG, which facilitated the diffusion of the drugs. The results were consistent with both pathogens. However, when comparisons were made between each AZG formulation, no considerable difference in antibacterial activity was found. The inhibition zone was absent in the blank niosomal gel samples comparable to the negative control, which was phosphate buffer. *Aa.* and *Pg.* are both recognized as major periodontal pathogens associated with severe forms of periodontitis [52]. Their infiltration into the sub-epithelium and connective tissue causes a persistent infection that is problematic for conventional treatment. Even scaling and root planing, the gold standards of periodontitis treatment, cannot remove all residing pathogens. The local delivery of antibiotics could eliminate the remaining bacteria. However, the formulation should exhibit a long retention time in the periodontal pocket and enhance transmucosal drug delivery.

Considering the results obtained, it is possible to conclude that after administration to the intricate gap of the periodontal pocket, AZG transformed into a gel state. The hydrogel matrix of P407–HA gradually eroded. AZM-loaded niosomes were released from the matrix by means of diffusion and erosion for more than 72 h. AZM should be delivered to the periodontal pocket and permeated to the surrounding periodontal tissues through the advantages provided by niosomes, to eliminate residing periodontal pathogens. Based on overall performance, AZG9 would be the most desirable formulation for periodontitis treatment. The selection of AZM as a model drug, along with HA as an additive, was considered on account of the anti-inflammatory properties of AZM and the wound healing properties of HA. These effects are advantageous for periodontitis treatment and will be further investigated in our upcoming study.

## 4. Conclusions

The primary outcome of this study was to invent a desirable formulation suitable for injection into the periodontal pocket for adjunctive periodontitis treatment. The developed AZG based on P407–HA interactions and AZM-loaded niosomes provided acceptable properties, such as ease of administration and sustained drug release with enhanced drug permeation, and improved the bioavailability of AZM in periodontal tissue. The invented formulations were effective in eliminating pathogenic bacteria. Therefore, within the limits of this study, AZG exhibited potential efficiency for periodontitis treatment, which should be further investigated in future research.

## Figures and Tables

**Table 1:** Composition of thermoresponsive azithromycin-loaded niosome gels (AZG).

Formulation	Niosomes of Azithromycin		Thermoresponsive Gel Compositions	
	AZM	S60:CHL	P407	HA
	(% *w*/*v*)	(Molar Ratio)	(% *w*/*v*)	(% *w*/*v*)
AZG1	1	3:3	17	0.2
AZG2	1	3:3	17	1.1
AZG3	1	3:3	17	2.0
AZG4	1	3:3	18	0.2
AZG5	1	3:3	18	1.1
AZG6	1	3:3	18	2.0
AZG7	1	3:3	19	0.2
AZG8	1	3:3	19	1.1
AZG9	1	3:3	19	2.0

**Table 2:** Physicochemical properties of AZG formulations (mean ± SD, n = 3).

Physical Properties	Gelation Temperature (°C)	Gelation Time (seconds)	pH	Drug Content (%)	Viscosity (cps)	
					4 °C	37 °C
Formulation						
AZG1	40.33 ± 0.06	227.00 ± 3.61	6.91 ± 0.02	93.86 ± 0.81	2.24 ± 0.25	162.77 ± 5.75
AZG2	36.60 ± 0.10	171.67 ± 3.51	6.90 ± 0.03	94.02 ± 1.28	29.86 ± 0.44	194.20 ± 10.14
AZG3	36.23 ± 0.15	87.33 ± 3.06	6.92 ± 0.04	93.90 ± 1.18	124.67 ± 13.51	216.07 ± 11.47
AZG4	34.20 ± 0.10	200.67 ± 3.05	6.89 ± 0.01	94.38 ± 0.56	2.60 ± 0.52	189.03 ± 4.28
AZG5	32.43 ± 0.38	125.33 ± 2.52	6.89 ± 0.01	93.87 ± 1.23	36.98 ± 4.43	195.10 ± 6.30
AZG6	33.10 ± 0.10	85.00 ± 4.58	6.90 ± 0.01	94.49 ± 0.81	125.30 ± 6.85	241.53 ± 9.56
AZG7	32.67 ± 0.15	173.33 ± 3.06	6.90 ± 0.02	93.09 ± 0.94	2.55 ± 0.30	218.97 ± 12.62
AZG8	29.60 ± 0.20	107.00 ± 3.00	6.90 ± 0.02	93.37 ± 0.92	34.73 ± 3.23	247.23 ± 13.21
AZG9	27.83 ± 0.55	68.00 ± 2.00	6.92 ± 0.01	93.64 ± 1.37	143.53 ± 15.55	283.77 ± 3.75

**Table 3:** Injectability and textural properties of AZG (mean ± SD, n = 3).

Mechanical Properties	Injectability (N)	Texture Profile Analysis		
		Hardness (mN)	Springiness (Ratio)	Resilience (Ratio)
Formulation				
AZG1	0.78 ± 0.01	249.80 ± 6.90	0.25 ± 0.01	0.002 ± 0.00
AZG2	0.94 ± 0.01	418.89 ± 18.60	0.24 ± 0.00	0.002 ± 0.00
AZG3	1.26 ± 0.06	554.96 ± 1.04	0.24 ± 0.00	0.003 ± 0.00
AZG4	1.24 ± 0.03	378.39 ± 4.14	0.25 ± 0.00	0.002 ± 0.01
AZG5	1.32 ± 0.03	472.05 ± 14.37	0.24 ± 0.00	0.002 ± 0.00
AZG6	1.50 ± 0.04	562.11 ± 0.03	0.24 ± 0.01	0.002 ± 0.01
AZG7	1.13 ± 0.03	400.60 ± 5.85	0.25 ± 0.00	0.002 ± 0.00
AZG8	1.55 ± 0.41	508.22 ± 12.91	0.24 ± 0.00	0.002 ± 0.00
AZG9	1.94 ± 0.04	617.29 ± 14.31	0.24 ± 0.00	0.003 ± 0.01

**Table 4:** Kinetic model fitting of AZG formulations.

Kinetic Models	Zero-Order		First-Order		Higuchi		Korsmeyer–Peppas		
	*K_0_*	*r^2^*	*K_1_*	*r^2^*	*K_H_*	*r^2^*	*K_KP_*	*n*	*r^2^*
Formulation									
AZG3	2.699	0.964	0.032	0.982	7.756	0.944	4.797	0.741	0.998
AZG6	2.600	0.953	0.030	0.975	7.505	0.952	4.905	0.715	0.998
AZG8	3.186	0.954	0.039	0.980	9.191	0.950	5.972	0.718	0.998
AZG9	2.567	0.938	0.030	0.963	7.452	0.963	5.211	0.681	0.999

*K_0_*, *K_1_*, *K_H_*, *K_KP_* are equation parameters of zero-order, first-order, Higuchi, Korsmeyer–Peppas, respectively. The n values are release exponents of Korsmeyer–Peppas equation.

## Data Availability

Not applicable.
